# Centenarians, semi and supercentenarians, COVID-19 and Spanish flu: a serological assessment to gain insight into the resilience of older centenarians to COVID-19

**DOI:** 10.1186/s12979-024-00450-3

**Published:** 2024-06-27

**Authors:** Claudia Maria Trombetta, Giulia Accardi, Anna Aiello, Anna Calabrò, Calogero Caruso, Mattia Emanuela Ligotti, Serena Marchi, Emanuele Montomoli, Martin Mayora Neto, Nigel Temperton, Giuseppina Candore

**Affiliations:** 1 Department of Molecular and Developmental Medicine, University of Siena, Siena, Italy; 2 VisMederi Research srl, Siena, Italy; 3 Laboratory of Immunopathology and Immunosenescence, Department of Biomedicine, Neurosciences and Advanced Diagnostics, University of Palermo, Palermo, Italy; 4 VisMederi srl, Siena, Italy; 5 Viral Pseudotype Unit, Medway School of Pharmacy, University of Kent, Kent, UK; 6 Present Address: Department of Research, ISMETT-IRCCS Mediterranean Institute forTransplants and Highly Specialized Therapies, Palermo, Italy

**Keywords:** Centenarians, COVID-19, Immune Response, Inflamm-ageing, Neutralising antibodies, Semi-supercentenarians, Spanish flu, Supercentenarians

## Abstract

**Background:**

Although it is well known that the older people have been the most susceptible to COVID-19, there are conflicting data on the susceptibility of centenarians. Two epidemiological study have shown that older centenarians (> 101 years old at the time of the 2020 pandemic peak) are more resilient than the remaining centenarians, suggesting that this resilience might be linked to the 1918 Spanish Flu pandemic. To gain insight into this matter, specifically whether the resilience of older centenarians to SARS-CoV-2 infection is linked to the Spanish Flu they had been affected by, we conducted a retrospective serological study. This study examined serum samples from 33 centenarians, encompassing semi- (aged > 104 < 110 years, *N* = 7) and supercentenarians (aged > 109 years, *N* = 4), born between 1905 and 1922, against both SARS-CoV-2 and 1918 H1N1 pseudotype virus.

**Results:**

Anamnestic and laboratory data suggest that SARS-CoV-2 infection occurred in 8 centenarians. The infection appeared to have been asymptomatic or mild, and hospitalization was not required, despite 3 out of 8 being between 109 and 110 years old. The levels of anti-spike antibodies in centenarians infected and/or vaccinated were higher, although not significantly, than those produced by a random sample of seventy-year-old individuals used as controls. All centenarians had antibody levels against the 1918 H1N1 virus significantly higher (almost 50 times) than those observed in the quoted group of seventy-year-old subjects, confirming the key role in maintaining immunological memory from a priming that occurred over 100 years ago. Centenarians whose blood was collected prior to the pandemic outbreak demonstrated neutralising antibodies against the 1918 H1N1 virus, but all these subjects tested negative for SARS-CoV-2.

**Conclusion:**

This retrospective study shows that older centenarians are quite resilient to COVID-19, as they are capable of producing good levels of neutralising antibodies and experiencing mild or asymptomatic disease. This could be attributed to the 1918 Spanish flu pandemic through mechanisms other than the presence of cross-reactive antibodies between the 1918 H1N1 virus and SARS-CoV-2. Another possibility is that the association is purely temporal, solely correlated with the advanced age of resilient centenarians compared to those born after 1918, since older centenarians are known to have better control of immune-inflammatory responses.

**Supplementary Information:**

The online version contains supplementary material available at 10.1186/s12979-024-00450-3.

## Background

The 1918 influenza pandemic and COVID-19 pandemic, respectively due to H1N1 and SARS-CoV-2 viruses, are among the most disastrous infectious disease emergencies of contemporary times. Although caused by unrelated viruses, the two pandemics are nevertheless similar in their clinical and pathological characteristics [1], but not in their epidemiological ones (see below).

During the COVID-19 pandemic, children and adolescents acquired SARS-CoV-2, often without manifesting serious symptoms. Likely explanations include regular exposure to seasonal coronaviruses, leading to the existence of cross-reactive antibodies, and concurrent clearance with other viral infections. Additional age-related factors may include very frequent immunizations and resulting trained immune responses, as well as a broader memory T cell repertoire compared to older individuals who, due to immune ageing and inflamm-ageing, were the age group with the highest mortality [2–4].

Specific age-related Spanish flu mortality followed, instead, a W-shaped curve characterized by high mortality in infants and young children, as well as in older people, with a third peak of mortality in individuals aged 15 to 30 years. Those over the age of thirty could have been protected by pre-existing cross-immunity likely due to an H1 flu virus that was in circulation in 1889 [1, 5]. Regarding infant mortality, a so-called honeymoon period was recognized, as mortality rates decreased after the first two years of life in 4–12 years old children, only to increase again in later childhood. This epidemiological pattern is consistent with host-pathogen coadaptation [5, 6].

Coming back to COVID-19 older people mortality, there are conflicting findings in the literature [7]. However, this quoted review revealed the following: (a) Consistent with evidence indicating that women tend to outlive men even during severe famines and epidemics, women exhibit greater resilience [8], although conflicting data exist regarding female centenarians in Northern Italy [9]; (b) Due to their frailty [10], centenarians, on the whole, generally succumb to COVID-19 at rates similar to or greater than other older individuals [11–13], with the exception of Japan, where their numbers continued to rise due to general precautions against viral infections, such as social distancing [14]; (c) During the initial wave of the pandemic in 2020, before introduction of vaccines, “older” centenarians (> 101 years old) exhibited greater resilience to COVID-19 than “younger” ones (< 102 years old) [11, 15].

The Belgian study highlighted that in 2020 the mortality rate among centenarians born after August 1, 1918, in Belgium was higher compared to older centenarians [15]. The Sicilian study, using mortality data from 2019 as a control, calculated the crude excess mortality between 2019 and 2020 for centenarians born after 1918 and those born before 1919. In 2020, there was a 61% excess mortality for centenarians born after 1918 and thus aged 100 or 101 years, while there was no excess mortality for centenarians born before 1919 and thus aged equal to or older than 102 years [11]. Both studies were conducted using demographic data published by the respective demographic institutions [11, 15].

This is an apparent paradox, as older centenarians are inherently more fragile than younger ones [10]. The temporal coincidence between the outbreak of the Spanish flu pandemic (on August 1, 1918, in Belgium, while the month of the outbreak is unknown in Sicily) and the birth of cohorts characterized by greater susceptibility to COVID-19 in 2020 suggests a connection between exposure to the 1918 H1N1 influenza pandemic and resistance to SARS-CoV-2 [11, 15]. Poulain et al. [15] have hypothesized that the lifelong persistence of cross-reactive immune mechanisms might have enabled centenarians exposed to the Spanish flu to overcome the threat of COVID-19 a century later.

To gain insight into this matter, namely whether the resilience of older centenarians (> 101 years old) to SARS-CoV-2 infection is linked to the Spanish Flu they had been affected by, we conducted a retrospective study. We tested serum samples from 33 Sicilian centenarians, including semi- (> 104 < 110 years old) and supercentenarians (> 109 years old), born between 1905 and 1922, against both SARS-CoV-2 and 1918 H1N1 pseudotype virus (PTV). Semi-supercentenarians and supercentenarians represent a highly selected population, comprised of individuals who have survived two world wars and numerous environmental and microbial challenges, including the Spanish flu [16]. Therefore, it is reasonable to infer that the immune systems of these individuals possess unique characteristics that contribute to their remarkable longevity [17–22].

## Results

Tables 1 and 2 present comprehensive data for the 33 centenarians included in this retrospective study, detailing information such as sex, date of blood draw, and age at the time of blood collection (expressed in years and months). The study cohort included four female supercentenarians, six female semi-supercentenarians, and one male semi-supercentenarian. Thirteen centenarians were born before 1919, and their blood samples were collected prior to the pandemic outbreak. In contrast, blood samples from another thirteen centenarians, also born before 1919, were drawn after the onset of the pandemic. Additionally, seven centenarians were born after 1918, and among these, blood samples were collected after the pandemic outbreak for six individuals. Furthermore, Table 2 documents the insights furnished by the offspring (or by caregivers) of the centenarians concerning SARS-CoV-2 infection and anti-COVID-19 vaccination.

Tables 1 and 2 also encompass the outcomes of the serological tests conducted. The ELISA test is employed to detect antibodies targeted against the nucleocapsid protein (NP), which are indicative of previous natural infection by SARS-CoV-2, since the mRNA vaccines do not elicit a response against this antigen, so much so that they can be used to distinguish individuals in a vaccinated population who have been previously exposed to the virus [23–27]. Then, neutralising antibodies are considered to be a correlate of protection, and the virus neutralisation (VN) assay with live viruses is currently considered the gold standard for assessing antibody-mediated protection in both naturally infected and vaccinated subjects [28, 29]. This assay enables the identification of neutralising antibodies specifically directed against the Spike (S) protein of the virus [30], thus conferring functionality in averting viral entry into cells. The results of the test to quantify neutralising antibodies against H1N1 PTV are also presented. Lastly, the Table 2 highlights the results/data of centenarians born before 1919 and the remaining ones separately.

**Table 1:** Data for centenarians born before 1919, whose blood was drawn before pandemic outbreak

Sex- Date of Birth	Date of blood draw	Age at the time of blood draw	Anti-H1N1 pseudotype virus values (neutralization assay)
1.F1913	10/07/2017	104.4	17,079
2.F1905	27/07/2017	111.8	8919
3.F1911	27/07/2017	105.6	14,410
4.F1916	24/11/2017	101.0	226,208
5.F1917	20/12/2017	100.2	22,138
6.M1914	29/11/2017	103.1	39,347
7.F1915	25/01/2018	102.2	3119
8.M1916	15/05/2018	102.4	4253
9.F1918	15/05/2018	100.2	2831
10.F1916	26/11/2018	102.1	26,221
11.F1914	22/01/2019	104.7	38,060
12.F1916	08/10/2019	103.2	4644
13.M1912	06/02/2020	108.1	28,166

In all samples both IgG vs. NP of SARS-CoV-2 (ELISA) and Titres anti- SARS-CoV-2 (neutralization assay), were negative

**Table 2:** Data for centenarians whose blood was drawn after pandemic outbreak

Sex- Date of Birth	Date of blood draw	Age at the time of blood draw	SARS- CoV-2 Infection	Number of vaccines doses and date of the last dose	IgG vs. NP of SARS-CoV-2 (ELISA)	Titres anti- SARS-CoV-2 (neutralization assay)	Anti-H1N1 pseudotype virus values (neutralization assay)
			*Centenarians born before 1919*				
14.F1909	13/07/2020	111.3	NO	N.A	Neg.	Neg.	37,455
15.F1913	24/09/2020	107.0	NO	N.A	Neg.	Neg.	1838
16.F1910	01/04/2021	110.3	N.R	N.R	Neg.	Neg.	22,928
17.F1914	27/05/2021	107.4	N.R	N.R.	Neg.	226	23,863
18.F1915	17/06/2021	105.6	N.R	N.R.	Pos.	2560	3006
19.F1917	17/06/2021	103.6	N.R	N.R.	Pos.	7241	5062
20.F1912	08/07/2021	109.5	NO	YES	Pos.	10	30,601
21.M1917	03/11/2021	104.1	N.R	N.R.	Neg.	Neg.	6747
22.F1914	11/11/2021	107.2	NO	NO	Pos.	3620	5544
23.F1912	24/3/2022	110.2	NO	NO	Pos.	Neg.	3487
24.F1918	16/6/2022	103.9	N.R	YES 4–19/4/22	Neg.	80	61,543
25.F1918	07/12/2022	104.3	NO	YES 3	Neg.	640	73,999
26.F1913	20/12/2022	109.0	YES	YES 2–03/01/22	Pos.	226	96,216
			*Centenarians born from 1919 onwards*				
27.F1919	08/10/2019	100.2	NO	NO	Neg.	Neg.	3776
28.M1919	28/07/2021	101.9	N.R.	N.R.	Neg.	Neg.	4708
29.F1919	15/10/2021	102.5	N.R.	N.R.	Pos.	7241	5736
30.F1920	21/02/2022	101.4	N.R.	YES 3–01/09/21	Neg.	20	5141
31.M1921	07/04/2022	100.5	N.R.	YES 3–17/02/22	Neg.	1280	39,052
32.F1920	12/10/2022	101.9	NO	YES 3 − 02/11/21	Neg.	640	3027
33.F1922	9/11/2022	100.5	YES	YES 2	Pos.	5120	5736

N.R. Not reported because neither the offspring nor the caregivers were able to provide information on the matter

Regarding the clinical data of the centenarians reported in Table 2, it is noteworthy that three of them were considered survivors based on the classification of Evert et al., [31] because two of them had a clinical history of colon cancer [29 F,30 F] and the third of two previous myocardial infarctions [28 M]. As expected, the majority had cardiovascular problems treated with antihypertensive drugs, diuretics, and antiplatelet agents depending on their clinical picture. One centenarian (19 F) had debilitating sarcopenia with cognitive impairment, which was also present in another centenarian (14 F) who had a history of hospitalizations for recurring pneumonia. Finally, it is very interesting to note that the daughter of 26 F reported that her mother had suffered from diphtheria as a child and a severe form of the Spanish Flu (she showed the highest anti-H1N1 PTV titre).

Regarding SARS-CoV-2 infection, it should be noted that while COVID-19 occurrence was confirmed through positivity in the ELISA vs. NP test in two female centenarians (26 F and 33 F), in another 6 centenarians (18 F, 19 F, 20 F, 22 F, 23 F, 29 F), the latter test was positive, despite their offspring or caregivers not reporting a previous diagnosis of COVID-19, and in three of these centenarians, it was even denied (20 F, 22 F, and 23 F). In the first two subjects, the disease was symptomatic with high fever and respiratory symptoms, and it was diagnosed as COVID-19 based on the nasal swab test, whereas in the last six centenarians the course of the disease was asymptomatic or resembled a common upper respiratory syndrome, and therefore it was not diagnosed. In the medical history, 8 centenarians were reported to have been vaccinated against SARS-CoV-2 (20 F, 24 F, 25 F, 26 F, 30 F, 31 F, 32 F, 33 F) and all of them exhibited neutralising antibodies against the virus with a wide range of antibody titres, ranging from 1/10 to 1/5120. However, five centenarians with negative or unreported information on the disease and vaccination (17 F, 18 F, 19 F, 22 F, 29 F) had neutralising antibodies against the virus with a wide range of responses (from 1/226 to 1/7241). The sera of four of these were also positive in ELISA, thus suggesting a past infection. Finally, it is noteworthy that the geometric mean of neutralising antibodies titres against SARS-CoV-2 among the 13 positive centenarians was higher, although not significant, likely for the low sample size, compared to that of a group of 30 individuals in their seventies (591 *±* 9 vs. 178 *±* 7, P = N.S.). Given the small number of centenarians born from 1919 onwards, of which 4 were vaccinated, it did not make sense to compare their antibody levels with those of centenarians born before 1919.

All 33 centenarians had neutralising antibodies against the H1N1 PTV [32], and their levels were significantly higher compared to those found in the same group of 30 individuals in their seventies (12,201 + 3 vs. 247 + 3, *P* < 0.0001). All centenarians whose blood was collected prior to the outbreak of the pandemic (subjects 1–13 and 27) show neutralising antibodies against the 1918 H1N1 PTV but all these subjects tested negative for SARS-CoV-2 (neutralisation assay and ELISA).

## Discussion

Regarding the possible resilience of centenarians to COVID-19, our results indicate that SARS-CoV-2 infection occurred in 8 centenarians, as suggested by the positivity of the ELISA test against NP. In most cases, the infection appeared to have been asymptomatic or mild, and hospitalization was not required, despite 3 out of 8 individuals being between 109 and 110 years old. It is important to note, however, that as reported by Lio et al. [33] and Caruso et al. [7], there are anecdotal data suggesting potential resilience to COVID-19 in centenarians, despite surveys suggesting that overall centenarians do not exhibit greater resilience than other older individuals [11–13]. Overestimating these anecdotal data may reflect a cognitive bias, as it naturally emphasizes survival rather than COVID-19 mortality. It is worth noting that most observations involve “older” female centenarians (> 101 years old) who were infected with SARS-CoV-2 in 2020 or the early months of 2021 and either recovered spontaneously or after a short hospitalization [7].

Further interesting results have been obtained from the study of anti-S neutralising antibodies. A study conducted on centenarians residing in long-term care facilities had already demonstrated that an old immune system is still capable of producing an antibody response to SARS-CoV-2 infection, and that the antibodies produced had neutralising abilities [34]. Our data confirmed this and additionally showed that the levels of produced antibodies were slightly higher than those produced by seventy-year-old individuals, although the difference was not significant.

As expected, all centenarians in the study had high levels of antibodies against H1N1 PTV, significantly higher than those observed in a group of seventy-year-olds. This underscores the crucial role of maintaining immunological memory from a priming event that occurred over a century ago. Previously, Yu et al. [35] investigated 32 individuals, aged between 91 and 101 years, who were born in or before 1915 (thus, aged 2 to 12 years in 1918), demonstrating their seropositivity to the 1918 virus (with a mean titre of 1/152), nearly nine decades following the pandemic. Among the 8 donor samples tested, seven showed the presence of circulating B cells secreting antibodies binding to the 1918 haemagglutinin. The study unveiled that survivors of the 1918 influenza pandemic harbour highly effective, neutralising antibodies against H1N1, and that B cells activated in response to viral infections, or their offspring, persist throughout the lifespan of the host, even after nine decades or more since exposure. Overall, these findings, along with our data, suggest that exposure to antigenically related viruses during the early decades of the 20th century may have contributed to the subject ability to maintain long-lived plasma cells (or to continuously produce them by B cells) capable of secreting antibodies against H1N1. Indeed, the 1918 pandemic eventually evolved into a less lethal annual seasonal pattern. The viral descendants of the “founder” virus from 1918 continue to circulate today as seasonal influenza viruses; subsequent pandemics in 1957, 1977, 2009, and subsequent years all resulted from genetic updates of the 1918 virus through mutational mechanisms [1, 36]. Therefore, the significant difference in antibody levels between centenarians and seventy-year-olds (almost 50 times) may also, in part, be attributed to the fact that seventy-year-olds have never been exposed to the H1N1 virus of the pandemic, but rather to its descendants with various mutations.

Furthermore, centenarians whose blood was collected prior to the outbreak of the pandemic showed evidence of an immune response to the 1918 H1N1 PTV but not to SARS-CoV-2. This data clearly indicates that there is no antigenic cross-reactivity between the two viruses in neutralisation tests. Therefore, the mechanisms underlying the greater resilience to COVID-19 in older centenarians born before 1919 cannot be explained by the presence of antigenic cross-reactivity between the two viruses. It is not known, however, if SARS-CoV-2 possesses cross-reactive antigens with the circulating coronaviruses from those years, potentially justifying an efficient immune response to SARS-CoV-2.

On the other hand, studies conducted in Japan, Sweden, Taiwan, and the USA have clearly demonstrated the long-term effects of prenatal exposure to the Spanish flu pandemic on various aspects such as height, level of education, physical abilities, depression, diabetes, and the incidence of renal, respiratory, and cardiovascular diseases among those exposed. Furthermore, exposed cohorts had excess all-cause mortality attributed to increased noncancer mortality [37–44]. Therefore, we could argue the possibility of the virus or pandemic stress also impacting the immune system, potentially leading to its enhanced resilience (see the reported cancer resistance in 39) in both surviving foetuses and children [7].

Another potential explanation concerns the possibility that the association between resistance to COVID-19 and the Spanish flu, observed in Belgian and Sicilian studies in centenarians born before 1918 during the epidemic peak of 2020 [11, 15], might be only temporal. This could indicate a correlation solely with the older age of resilient centenarians compared to those born after 1918, keeping in mind that the majority of older centenarians, including those in the present study, are female (see below). As stated in the background, in older people, COVID-19 is more severe and lethal due to immune ageing and inflamm-ageing. Moreover, the disease is more severe and lethal in men than in women [3, 7]. The degree of immune-inflammatory responses differs between men and women and persists throughout life [7, 8, 45]. Besides sex hormones, sex chromosomes play a role, as several immune genes are found on the X chromosomes. X-chromosome inactivation silences one X chromosome in the majority of XX cells, equalizing the expression of X-linked genes to that in XY cells. However, not all genes are silenced; those that evade X inactivation are believed to play a role in certain immune responses [8, 46] Generally, women have a stronger adaptive immune response (especially humoral), while men have a stronger natural immune response, i.e., an inflammatory one [7, 8, 45, 47]. Excess mortality observed in 27 European countries during the winter circulation of respiratory pathogens from 2016 to 2020 was higher in males compared to females. This pattern was particularly evident during influenza epidemics and the SARS-CoV-2 pandemic, indicating that sex and gender differences are common to all, or at least respiratory, infections [48]. The higher inflammatory response in men further contributes to high excess mortality from infectious diseases such as COVID-19, where the cytokine storm plays a key role [3, 45]. These differences persist in centenarians, since women immune parameters decline more slowly with ageing [45]. Both sexes experience increased pro-inflammatory states with age due to greater chromatin accessibility for inflammation-related genes, but it is more pronounced in men [47]. Despite this, the harmful effects of inflamm-ageing can be mitigated in centenarians through mechanisms such as the secretion of certain microRNAs, Interleukin-19, and increased regulatory T cells [49–51]. Interestingly, a preliminary report [52] calculated both the INFLA-score and the systemic inflammation response index (SIRI), two composite indices summarizing the effect of multiple serum and cellular inflammatory biomarkers [53, 54], to measure the level of inflamm-ageing, which showed a significant increase with age. However, no statistical difference was observed when analysing the values for semi- and supercentenarians compared to adults. Therefore, older centenarians have efficient mechanisms for controlling inflammation. Recent studies of lymphocyte subsets in semi- and supercentenarians suggest, then, that immune system ageing changes should be considered a specific adaptation that enables older centenarians to successfully cope with a lifetime of antigenic challenges and achieve extreme longevity [20–22]. Overall, the data demonstrate that older centenarians have better control of immune-inflammatory responses, which can allow for good control of SARS-CoV-2 infection, potentially resulting in asymptomatic cases.

Our study has several limitations. The number of enrolled centenarian subjects was relatively small, but it is important to consider that semi and supercentenarians are relatively rare (the ratio of supercentenarians to centenarians is 1/1000) [16]. Moreover, in a retrospective study where centenarians were recruited either before or during the pandemic, the case series would not be homogeneous if new participants are recruited after the end of the pandemic [55], due to the different time frame. The gender distribution was not balanced, because it reflected the female/male ratio among Italian centenarians, which is 85% vs. 15%. (for sex differences between centenarians, semi- and supercentenarians, and their number, see Supplementary file). Control sera were randomly selected solely based on the age range, without knowledge of the history of exposure to the virus or the vaccine, to ensure greater suitability as random controls. We did not evaluate other branches of immunity, such as T cell responses, despite their role in controlling SARS-CoV-2 [56]. Finally, it should be noted that the samples were collected from Southern Italy, so they may not be representative of the entire Caucasoid population.

## Conclusions

This retrospective investigation shows that older centenarians display considerable resistance to COVID-19, as they are capable of generating high levels of neutralising antibodies, and the infection was mild or asymptomatic. This resilience may be attributed to the 1918 Spanish flu through mechanisms other than the existence of cross-reactive antibodies between the 1918 H1N1 influenza virus and SARS-CoV-2. Another possibility is that the association is purely temporal, linked only to the greater age of resilient centenarians compared to those born after 1918, as older centenarians are recognized for having better control over immunoinflammatory responses.

## Materials and methods

### Study cohort

The subjects participating in the “Discovery of molecular and genetic/epigenetic signatures underlying resistance to age-related diseases and comorbidities (DESIGN, 20157ATSLF) project”, funded by the Italian Ministry of Education, University, and Research, were examined for the present investigation. The detailed study design and participant recruitment have been previously outlined [57]. For the present study, a total of 33 Sicilian participants (28 females and 5 males) aged between 100 and 111 years, enrolled between 2017 and 2022, were included. For their age validation see supplementary file. Centenarians were excluded from enrolment if diagnosed with chronic or acute diseases such as neoplastic and autoimmune diseases, as well as severe dementia. Another exclusion criterion was the use of immunomodulatory drugs within the previous six months. The subjects participated voluntarily, and written informed consent was obtained from all participants or their offspring.

Centenarians underwent venepuncture after a 12-hour fasting period in the morning (10 a.m.). Blood was collected in specific tubes containing EDTA or no additives. Sera were immediately frozen at -80 °C and subsequently, shipped, in dry ice, to the laboratory involved in the study. All serum samples underwent testing by VN assay with live (SARS-CoV-2) [30] and H1N1 PTV [32] and by ELISA for the detection of antibodies against the NP of SARS-CoV-2, indicative of previous infection [23–27].

As a control cohort, a total of 30 human serum samples from the Italian general population were used, selected based on the age range (70–79 years). These samples were anonymously collected in 2022 in the Apulia region (Southern Italy) as residual sera of routine medical checks. For each sample, only the date of collection and the subject age and sex (13 women and 17 men) were recorded.

### Determination of antibodies against the nucleocapsid protein of SARS-CoV-2

All samples were tested by commercial ELISA (Aeskulisa^®^ SARS-CoV-2 NP IgG, Aesku.Diagnostics, Wendelsheim, Germany) for the detection of IgG antibodies against the NP, which are indicative of previous natural infection [23–27]. In accordance with the manufacturer instructions, quantitative analysis was performed by using a 4-parameter logistic standard curve obtained by plotting the optical density (OD) values measured for 4 calibrators against their antibody activity (U/ml) using logarithmic/linear coordinates. The antibody activities of the samples were quantified from the OD values by using the curve generated, and were considered positive if > 12 U/ml.

### SARS-CoV-2 virus neutralisation assay

The VN assay, considered the gold standard for assessing antibody-mediated protection in both naturally infected and vaccinated subjects [28, 29], was performed as previously reported [58]. Briefly, serum samples were heat-inactivated for 30 min at 56˚C and, starting from 1:10 dilution, were mixed with an equal volume of ancestral wild-type SARS-CoV-2 2019 virus (2019-nCov/Italy-INMI1 strain, purchased from the European Virus Archive goes Global, EVAg, Spallanzani Institute, Rome) viral solution containing 100 Tissue Culture Infective Dose 50% (TCID_50_). After 1 h of incubation at room temperature, 100 µl of virus-serum mixture were added to a 96-well plate containing VERO E6 cells with 80% confluency. Plates were incubated for 72 h at 37˚C, 5% CO_2_ in humidified atmosphere, then inspected for presence/absence of cytopathic effect (CPE) by an inverted optical microscope. A CPE higher than 50% indicated infection. The VN assay titre was expressed as the reciprocal of the highest serum dilution showing protection from viral infection and CPE.

Samples with antibody titres equal to or greater than 10 are considered positive, while a titre of 5 indicates a negative sample, meaning no neutralising antibodies were detected. Serum samples were assayed in duplicate, and the presented results are the geometric mean titres.

### H1N1 pseudotype virus production

H1N1 PTV was produced in HEK293T/17 cells (ATCC CRL11268) pre-seeded in a T75 flask (Thermo) with approximately 2 × 10^6^ cells the day before transfection. Cells were then co-transfected with 1 µg of packaging lentiviral core p8.91, 1.5 µg of pCSFLW encoding Firefly Luciferase, 1 µg of A/South Carolina/1/1918 haemagglutinin phCMV1 plasmid (accession no AF117241.1) and 250 ng TMPRSS4 pCMV using FugeneHD (Promega) transfection reagent at a ratio of 1:3 DNA: Fugene in optiMEM (Gibco). The day after transfection, 1U of exogenous Neuraminidase (Sigma-Aldrich N2876) was added to the cell culture medium. PTVs were harvested at 48 h post transfection and supernatant filtered through a 0.45 μm acetate cellulose filter (Starlab) [59, 60].

### H1N1 PTV neutralisation assay

PTV neutralisation assays were performed as described [32]. Briefly, serum samples were diluted 1:20 in DMEM and serially diluted 2-fold in white 96-well F-bottom plates (Thermo). An equal number of PTVs were added to achieve ~ 1 × 10^6^ RLU per well apart from cell only control wells, and incubated for 1 h at 37 °C and 5% CO_2_, followed by addition of target HEK293T cells (20,000 cells/well). Plates were incubated for 48 h prior to lysis with Bright-Glo reagent (Promega).

Luminescence was measured using a GloMax luminometer (Promega) and IC_50_ values (half-maximal inhibitory concentration) were determined by non-linear regression using GraphPad Prism (v.9).

### Statistics

The geometric mean titres of the different neutralisation assays were compared between centenarians and sample controls by unpaired Student t test. Only p-values less than 0.05 were considered significant.

### Electronic supplementary material

Below is the link to the electronic supplementary material.

Supplementary Material 1

## Data Availability

To protect privacy, raw data supporting the findings of this study are available from the corresponding authors upon written reasonable request.

## Abbreviations

COVID-19: Coronavirus disease 2019
NP: nucleocapsid protein
PTV: Pseudotype virus
S: Spike
SIRI: Systemic inflammation response index
SARS-CoV-2: Severe acute respiratory syndrome coronavirus 2
VN: Virus neutralisation
