# Supplementary material for: Centenarians, semi and supercentenarians, COVID-19 and Spanish flu: a serological assessment to gain insight into the resilience of older centenarians to COVID-19
Source: Immun Ageing. 2024 Jun 27;21:44. doi: 10.1186/s12979-024-00450-3 (PMC11210044; doi:10.1186/s12979-024-00450-3)
Supplement: Supplementary file 1 — Supplementary Material 1 [file 12979_2024_450_MOESM1_ESM.docx]

**SEX DIFFERENCES**

It is well known that the majority of centenarians are female. The percentage varies according to different scientific databases. For example, according to the Boston University database [1], 85% of centenarians are women and 15% are men. Among supercentenarians (110+), the female prevalence may increase to about 90%. According to the Italian Institute of Statistics [2], as of January 1, 2021, there were 17,177 residents in Italy aged 100 years and over. In 83% of cases, these were women. There were 1,111 residents who, as of January 1, 2021, had reached and exceeded the age of 105, about 9 out of 10 of whom were women. As of January 1, 2021, 17 women had reached and exceeded the age of 110 (supercentenarians). Moreover, according to the website Supercentenarians of Italy [3], there are currently 29 supercentenarians (over 110 years old) living in Italy, of which only one is male, and 205 semi-supercentenarians (limited to those aged over 107 years old), of which 15 are male, with a ratio of one male for nearly every 13 females (12.6667). The website is managed by Mr. Alessandro Delucchi, a representative of the European Supercentenarian Organisation and of Longevity Quest, data provider to the Buck Institute for Research on Aging.

1. Centenarian Statistics - Boston University Medical Campus <https://www.bumc.bu.edu/centenarian/statistics/> accessed June 10, 2024
2. ISTAT. <https://www.istat.it/it/files//2022/06/STAT-TODAY_CENTENARI-2021.pdf>
3. Supercentenari d’Italia <https://www.supercentenariditalia.it/persone-viventi-piu-longeve-in-italia>

**AGE VALIDATION**

**For all recruited centenarians, the data from their Identity Cards and Tax Codes, as well as those of their offspring caregivers, were checked for consistency with each other and with the reported marriage date along with other family data. It is noteworthy that the identity card of semi-supercentenarians is validated by** the Italian Institute of Statistics **(ISTAT) semi-supercentenarians (SSC) survey [1]:** The SSC survey collects data about alive or deceased resident population aged 105 and more with the aim of providing a longitudinal database of this population group.  The initial data source for the validation process is an administrative source. That is the National Register of the Resident Population (ANPR). All municipalities that have at least one semi-super centenarian or supercentenarian resident are contacted to provide a birth or death certificate and to collect additional demographic information.  For those people still alive at age 105 a yearly based follow-up is done until death, which is then included in the database (date of death): as long as the individual remains alive, the validation process never stops and each single year ISTAT proceeds to “re-validate” backward the data observed in the past. Therefore, the data quality check for the SSC’s database is performed retrospectively day by day. Moreover, we identified and recruited centenarians aged 108+ years through the website managed by Mr. Alessandro Delucchi [2], a representative of the European Supercentenarian Organisation and of Longevity Quest, data provider to the Buck Institute for Research on Aging. The age is validated by obtaining 3 documents in addition of Identity Card and Tax code: Original birth certificate or an equivalent document (e.g., baptism certificate), certificate of existence at an intermediate age after 20 years and before 100 years, approximately, certificate of existence for a supercentenarian. Obviously, all documents must be consistent with each other and linked through the numerical details of the historical certificates provided.

1. ISTAT <http://dati.istat.it/Index.aspx?QueryId=57350&lang=en>
2. Supercentenari d’Italia <https://www.supercentenariditalia.it/persone-viventi-piu-longeve-in-italia>
